# Supplementary material for: Empirical methods for controlling false positives and estimating confidence in ChIP-Seq peaks
Source: BMC Bioinformatics. 2008 Dec 5;9:523. doi: 10.1186/1471-2105-9-523 (PMC2628906; doi:10.1186/1471-2105-9-523)
Supplement: Additional File 1 — A variety of html documents from the USeq web site detailing the available applications, their best usage, output file type descriptions, command line menus, etc. [file 1471-2105-9-523-S1.zip › USeqUserGuides/JavaDoc.zip!/JavaDoc/trans/anno/class-use/Region.html]

Uses of Class trans.anno.Region


|  |  |  |
| --- | --- | --- |
| **Overview** \| **Package** \| **Class** \| **Use** \| **Tree** \| **Deprecated** \| **Index** \| **Help** | |  |
| PREV   NEXT | **FRAMES**    **NO FRAMES**     **All Classes** |  |


---


## **Uses of Class trans.anno.Region**

| Packages that use Region | |
| --- | --- |
| **trans.anno** | For associating intervals, binding regions, and binding peaks with GFF annotation. |

Uses of Region in trans.anno

| Methods in trans.anno that return Region | |
| --- | --- |
| `Region` | `RandomRegions.findRandomRegion(int minimumLength)`             Attempt to find a random Region of the appropriate minimum length. |
| `static Region[]` | `Region.loadBinaryCoordinates(java.io.File file)`             Reads binary chrom start stop file into Region[] |
| `static Region[]` | `RandomRegions.loadBinaryCoordinatesAsRegionArray(java.io.File file)`             Reads binary chrom start stop file into Region[] |
| `static Region[]` | `RandomRegions.loadWriteBinaryCoordinates(java.io.File regionsFile)`             Looks for a binary version of the file xxx.corr, if found loads binary, otherwise it parses the txt file and then writes the binary for future use. |
| `static Region[]` | `Region.loadWriteBinaryRegions(java.io.File regionsFile)`             Looks for a binary version of the file xxx.corr, if found loads binary, otherwise it parses the txt file and then writes the binary for future use. |
| `static Region[]` | `Region.parseRegions(java.io.File file)`             Parses a file for a tab delimited list of at minimum, chrom, start, stop, (optional) notes into a GenomicRegion[]. |

| Methods in trans.anno with parameters of type Region | |
| --- | --- |
| `int` | `Region.bpIntersectionSameChromosome(Region other)`             Assumes regions are on the same chromosome, and the end base is included, not interbase numbering. |
| `void` | `IntersectKeyWithRegions.calculateAndPrintStats(Region[] r)` |
| `int` | `RandomRegions.countLengths(Region[] rs)` |
| `boolean[][]` | `RandomRegions.fetchGCContent(Region[] r)`             Converts the Region[] sequences into boolean[]s, everything not g or c are recorded as false. |
| `int[]` | `RandomRegions.findRandomSegment(Region region, int length)`             Returns a random segment of the appropriate length within the region. |
| `int` | `IntersectKeyWithRegions.intersectsKey(Region region)`             Returns 1 if bpInt <= maxGap and the particular key hasn't been hit, 0 not in key, -1 hits a key that has already been counted. |
| `static void` | `RandomRegions.saveRegionsAsCoordinatesArray(java.io.File file, Region[] r)`             Writes the chrom start stop of a Region[] as a binary file. |
| `static java.util.HashMap` | `Region.splitByChromosome(Region[] sorted)`             Given a chromosome SORTED array of Regions returns a HashMap containing chromosome: sorted chromo specific Region[] |
| `static void` | `Region.writeBinaryCoordinates(java.io.File file, Region[] r)`             Writes the chrom start stop of a Region[] as a binary file. |

---


|  |  |  |
| --- | --- | --- |
| **Overview** \| **Package** \| **Class** \| **Use** \| **Tree** \| **Deprecated** \| **Index** \| **Help** | |  |
| PREV   NEXT | **FRAMES**    **NO FRAMES**     **All Classes** |  |


---
